# Supplementary material for: Manipulating virulence factor availability can have complex consequences for infections
Source: Evol Appl. 2016 Oct 24;10(1):91–101. doi: 10.1111/eva.12431 (PMC5192820; doi:10.1111/eva.12431)
Supplement: Supplementary file 1 [file EVA-10-91-s001.pdf]

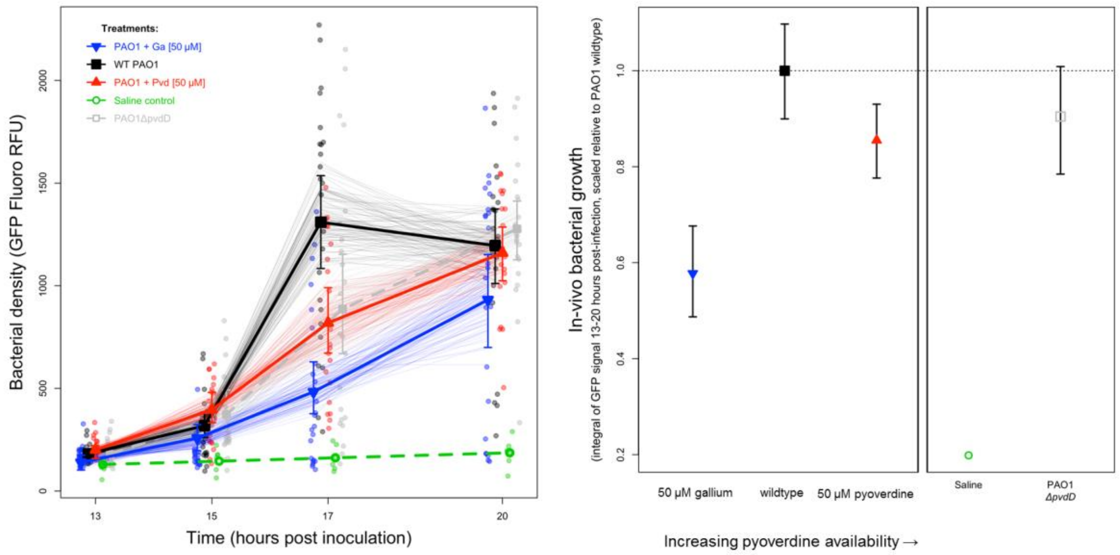

Figure S1. Growth trajectories of *P. aeruginosa* within *G. mellonella* larvae confirm that gallium and pyoverdine supplementation both significantly reduced bacterial growth compared to the unsupplemented wildtype (permutation test with 10,000 iterations: p =0.030). *In vivo* bacterial density was estimated from constitutively expressed GFP signal in host homogenates (points). This involved destructively sampling up to 96 larvae per treatment (~24 per time point; or n = 6 for the saline control). Because we were unable to track infections within an individual through time, we used bootstrap resampling of our observed data to generate replicated sets of estimated trajectories, a random sample of which are shown (faint lines). Symbols and error bars denote the medians, 2.5% and 97.5% quantiles from n=10,000 bootstrap-replicated datasets. We fitted splines to each trajectory and summarized the overall growth patterns using areas-under-curves. These resulting distributions of these growth integrals are given in the final plot (medians with 2.5% and 97.5% quantiles).

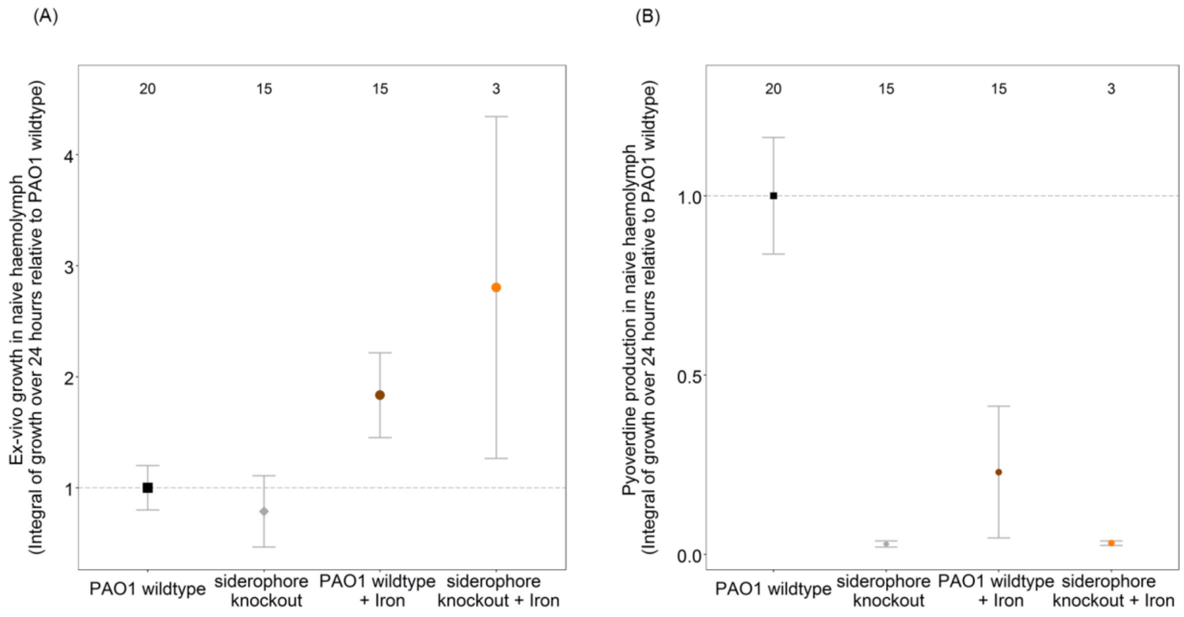

Figure S2 Growth (A) and pyoverdine production (B) of the wildtype strain and the pyoverdine-deficient mutant in naive haemolymph extracted from *G. mellonella* larvae. (A) The supplementation of 100 µM $FeCl_3$ to the haemolymph significantly increased the growth of both the wildtype strain and the pyoverdine-deficient mutant, demonstrating that iron is a growth-limiting factor in the host environment. The observed pyoverdine-production profiles confirmed this assertion (B). Specifically, the wildtype strain produced high amounts of pyoverdine in the unsupplemented haemolymph, but reduced its investment to baseline level when iron was added to the haemolymph. Numbers on top show sample size of each treatment.

Gene	Sequence (5`→ 3`)
pvdS (forward)	AGG AAG AAG GCC TGA ACG TG
pvdS (reverse)	CCT TGG CGA TGT CCT TCT GT
pvdA (forward)	TGT TCC ACC ACA GCC AGT AC
pvdA (reverse)	GGG TAG CTG TCG TTG AGG TC
toxA (forward)	AAA AGC GCT GGA GCG AAT GG
toxA (reverse)	GGG AAA TGC AGG CGA TGA CTG AT
prpL (forward)	TCT ACA ACA CCA CCC AGT GC
prpL (reverse)	TTG CCC TGC GAG TAC TTC TT
rpoD (forward)	GGG GAT CAA CGT ATT CGA GA
rpoD (reverse)	ATC GAT ATA GCC GCT GAG GA

S1 Table. Genes and primers used for qPCR. We studied four genes involved in pyoverdine-mediated signaling, which are *pvdS* (encoding the iron-starvation sigma factor), *pvdA* (coding for an enzyme critical for pyoverdine synthesis), *toxA* (coding for exotoxin A), and *prpL* (encoding protease IV). Primers were designed based on sequences from the *Pseudomonas* genome database (www.pseudomonas.com) using the Primer3Plus platform aiming for 200 bp amplicons.
